# Selection of suitable reference genes for quantitative real-time PCR gene expression analysis in *Salix matsudana* under different abiotic stresses

**DOI:** 10.1038/srep40290

**Published:** 2017-01-25

**Authors:** Yunxing Zhang, Xiaojiao Han, Shuangshuang Chen, Liu Zheng, Xuelian He, Mingying Liu, Guirong Qiao, Yang Wang, Renying Zhuo

**Affiliations:** 1 State Key Laboratory of Tree Genetics and Breeding, Chinese Academy of Forestry, Beijing 100091, China; 2 Key Laboratory of Tree Breeding of Zhejiang Province, The Research Institute of Subtropical of Forestry, Chinese Academy of Forestry, Hangzhou, Zhejiang 311400, China; 3 School of Architectural and Artistic Design, Henan Polytechnic University, Jiaozuo, Henan 454000, China; 4 College of Plant Protection, Yunnan Agricultural University, Kunming, Yunnan 650201, China

## Abstract

*Salix matsudana* is a deciduous, rapidly growing willow species commonly cultivated in China, which can tolerate drought, salt, and heavy metal stress conditions. Selection of suitable reference genes for quantitative real-time PCR is important for normalizing the expression of the key genes associated with various stresses. To validate suitable reference genes, we selected 11 candidate reference genes (five traditional housekeeping genes and six novel genes) and analyzed their expression stability in various samples, including different tissues and under different abiotic stress treatments. The expression of these genes was determined using five programs—geNorm, NormFinder, BestKeeper, ΔCt, and RefFinder. The results showed that *α-TUB2* (alpha-tubulin 2) and *DnaJ* (chaperone protein DnaJ 49) were the most stable reference genes across all the tested samples. We measured the expression profiles of the defense response gene *SmCAT* (catalase) using the two most stable and one least stable reference genes in all samples of *S. matsudana*. The relative quantification of *SmCAT* varied greatly according to the different reference genes. We propose that *α-TUB2* and *DnaJ* should be the preferred reference genes for normalization and quantification of transcript levels in future gene expression studies in willow species under various abiotic stress conditions.

Drought, salt, and heavy metal stresses are major abiotic factors that contribute to the risk of environment and affect forestry productivity worldwide^1^,^2^,^3^,^4^,^5^; however, plants need to thrive in adverse circumstances^6^. Plants with short growth cycles, such as *Arabidopsis thaliana*^7^, soybean^8^, sorghum^9^, jute^10^, *Sedum alfredii*^11^, rice^12^, and tobacco^13^, have been the focus of studies on the effects of various abiotic stresses, and a few studies have been performed on plants with long growth cycles under different stress conditions. Short growth cycle plants are limited by low biomass, while plants (especially woody plants) with high biomass and long growth cycles are more able to deal with severe abiotic stress conditions. Only a small number of reference genes have been reported in trees under drought, salt, and heavy metal stress conditions^14^,^15^,^16^,^17^,^18^.

The genus *Salix* (Salicaceae) contains more than 450 willow species worldwide; 275 of these species grow in China^19^,^20^,^21^,^22^. Willow species are used for energy production, afforestation, and greening due to their high biomass, rapid growth, and ability to adapt to different stress conditions^23^,^24^,^25^,^26^,^27^,^28^. *Salix matsudana* is a deciduous, rapidly growing willow species commonly cultivated in China, which can tolerate drought, salt, and heavy metal stresses^29^,^30^,^31^,^32^,^33^. Physiological and biochemical properties have been characterized in *S. matsudana*^34^,^35^. Meanwhile, some key genes have been identified to regulate stress response factors in stressed plants at the molecular level^36^,^37^,^38^. Understanding the expression patterns of key stress response genes will help elucidate the mechanisms involved in various stresses of *S. matsudana*.

Gene expression analysis has been applied to understand different kinds of biological processes^39^. Quantitative real-time polymerase chain reaction (qRT-PCR) is widely used for gene expression analysis due to its high sensitivity, accuracy, specificity, and reproducibility^40^,^41^,^42^. However, factors such as sample amount, RNA integrity, reverse transcription efficiency, and cDNA quality can significantly influence the reliability of the gene expression results^43^,^44^,^45^. To reduce the influence of these factors, internal reference genes are used to obtain accurate biologically meaningful expression values^46^; however, unstable reference genes can cause significant biases and misinterpretations of the expression data^47^,^48^. Actin (*ACT*) and *β*-tubulin (*β-TUB*) have been used as reference genes for qRT-PCR normalization in gene expression analysis in *S. matsudana* under salt and copper stresses^37^,^49^; however, a systematic study to validate reference genes has not been reported for *S. matsudana* under abiotic stresses. To obtain accurate expression data, it is necessary to select suitable reference genes for each plant species and to verify their stability under the specific experimental conditions of interest.

In this study, we determined the expression profiles of 11 candidate reference genes from *S. matsudana* in six different tissues and under three kinds of abiotic stresses. The 11 candidate genes were *ACT*, alpha-tubulin 1 (*α-TUB1*), alpha-tubulin 2 (*α-TUB2*), chaperone protein DnaJ 49 (*DnaJ*), E3 ubiquitin-protein ligase ARI8 (*ARI8*), F-box family protein (*F-box*), histone H2A (*H2A*), heat shock 70 kDa protein (*HSP 70*), glyceraldehyde-3-phosphate dehydrogenase (*GAPDH*), membrane-anchored ubiquitin-fold protein (*MUB*), and *β-TUB*. The transcriptome data of *S. matsudana* were used as the source of the potential reference genes (Unpublished data). The stabilities of the 11 reference genes were analyzed using five statistical algorithms—geNorm^43^, NormFinder^44^, BestKeeper^50^, ΔCt method^51^, and RefFinder, a web-based software^52^. The expression levels of the defense response gene *SmCAT* (catalase) as a target gene were assayed to verify the selected reference genes. The results will provide suitable reference genes for qRT-PCR normalization for accurate gene expression analysis in *S. matsudana* under different stress conditions.

## Materials and Methods

### Plant materials and stress treatments

Cuttings (approximately 10 cm long) from annual branches of *S. matsudana* were grown in hydroponics. Plants were supplemented with water containing 1/4 strength Hoagland^53^ solution on alternate days under normal conditions (25 °C, 16 h light/8 h dark). After 45 days of culture, groups of *S. matsudana* seedlings were subjected to different abiotic stresses in solutions containing 1/4 strength Hoagland solution at pH 6.0 as follows: drought (15% PEG 6000), salt (100 mM NaCl), and heavy metal (100 μM CdCl_2_). Untreated seedlings were used as the control. The roots of the treated plants were sampled at 0 h, 12 h, 24 h, 48 h, and 72 h. Tissues from the root, xylem, bark, stem, leaf, and flower were collected from the untreated plants. All the samples from three biological replicates were carefully harvested, immediately frozen in liquid nitrogen, and stored at −80 °C until total RNA extraction.

### Total RNA isolation and cDNA synthesis

Total RNA from each sample was isolated from approximately 0.1 g fresh root using a total RNA kit (NORGEN, Thorold, Canada) and treated with DNase I (TaKaRa, Dalian, China) to remove any genomic DNA contamination. The RNA concentration of each sample was determined using a NanoDrop-2000 spectrophotometer (Thermo, Wilmington, USA). Samples with a 260/280 ratio of 1.9–2.1 and a 260/230 ratio ≥2.0 were chosen to determine the quality and purity of the RNA preparations. The integrity of the purified RNA was checked by 1.0% (p/v) agarose gel electrophoresis. Subsequently, first-strand cDNA was synthesized in a 20-μL reaction mixture in an Invitrogen SuperScript First Strand Synthesis System (Invitrogen, Carlsbad, USA) following the manufacturer’s instructions, and stored at −20 °C until use.

### Screening of candidate reference genes and primer design

We identified 11 candidate reference genes and one target gene (Table 1) from the *S. matsudana* transcriptome data. Primers were designed based on the sequences the 11 genes using Primer3 (http://bioinfo.ut.ee/primer3-0.4.0/primer3/) with the following criteria: GC content 45–65%, optimal Tm 58–61 °C, primer length 18–22 bp, and amplicon length 120–220 bp (Table 1). The specificity of each selected primer pair was observed via standard RT-PCR using Premix Ex Taq (TaKaRa, Dalian, China), and each gene was verified by 2% agarose gel electrophoresis and sequenced to ensure its reliability.

### qRT-PCR

qRT-PCR amplification was performed in 96-well plates with a Applied Biosystems 7300 Real-Time PCR System (Applied Biosystems, CA, USA) using SYBR^®^ Premix Ex Taq™ (TaKaRa, Dalian, China). PCR reactions were prepared in 20 μL volumes containing: 2 μL of 50-fold diluted synthesized cDNA, 10 μL 2 × SYBR Premix Ex Taq™, 0.8 μL of each primer, 0.4 μL ROX reference dye (50×), and 6.8 μL ddH_2_O. The reactions comprised an initial step of 95 °C for 30 s, followed by 40 denaturation cycles at 95 °C for 5 s and primer annealing at 60 °C for 31 s. Next, the melting curves ranging from 60 °C to 95 °C were evaluated in each reaction to check the specificity of the amplicons. Biological triplicates of all the samples were used for the qRT-PCR analysis, and three technical replicates were analyzed for each biological sample. The threshold cycle (Ct) was measured automatically.

### Statistical analysis to determine the expression stability of the candidate reference genes

Standard curves were generated in Microsoft Excel 2013 to calculate the gene-specific PCR efficiency and the correlation coefficient from 5-fold series dilution of a mixed cDNA (flower, bark, and stem) template for each primer pair. The amplification plots, melting curves and sequencing peaks were shown in Figure S1a,b,c. The PCR amplification efficiency (*E*) and the correlation coefficient were calculated using the slope of the standard curve according to the equation *E* = [5^−1/slope^ − 1] × 100. Stabilities of the 11 selected reference genes were evaluated by four algorithms—geNorm, NormFinder, BestKeeper, and the ΔCt method. Finally, RefFinder (http://www.fulxie.0fees.us), a comprehensive evalution platform integrating the four above algorithms, ranked the overall stabilities of these 11 candidate genes. Pairwise variations based on the geNorm calculation were used to determine the optimal number of candidate reference genes for accurate normalization.

### Expression normalization of *SmCAT* gene based on different reference genes

The defense response gene *SmCAT* was selected as the target gene to measure the stabilities of the candidate reference genes by quantifying *SmCAT* expression levels in all the tested samples. *SmCAT* gene expression levels were normalized with the two most stable candidate reference genes (*α-TUB2* and *DnaJ*), as well as one of the least stable reference genes (*β-TUB*).

## Results

### qRT-PCR data for the candidate reference genes

The 11 selected candidate reference genes (Table 1) are orthologs of genes in *Salix purpurea*, for which the whole genome has been sequenced. The specificity and accuracy of the primers designed for the selected genes were determined by 2% agarose gel electrophoresis (Figure S2a), and further confirmed by a single peak in the melting-curve analysis (Figure S2b). The primer sequences, amplicon length, correlation coefficient, and PCR amplification efficiency are shown in Table 1. Furthermore, the qRT-PCR products were sequenced (File S1) to determine the accuracy of the 11 genes.

**Figure 1:**
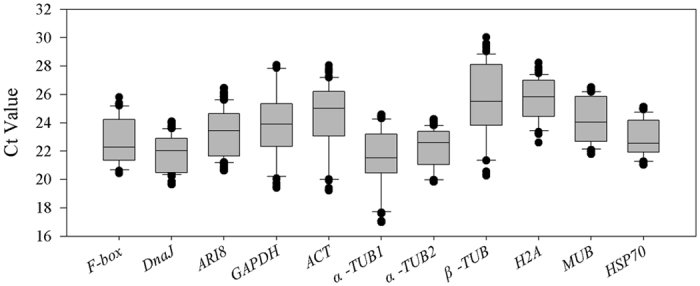
Expression levels of 11 candidate reference genes across all experimental samples.

To evaluate the stability of the 11 candidate reference genes at the transcript level under the three abiotic stress conditions, the gene expression levels were determined by the average Ct values, which varied from 17 to 30 (Fig. 1). According to the average Ct values of all the samples, *α-TUB1* was the most abundantly expressed gene, followed by *DnaJ, α-TUB2*, and *F-box*, while *H2A* was the least abundantly expressed gene, followed by *β-TUB, ACT* and *MUB*.

### Analysis of gene expression stability

Expression stabilities of the 11 candidate reference genes were determined using geNorm, NormFinder, ΔCt, and BestKeeper, and their overall stabilities were ranked by RefFinder across all the stress treatments and tissue samples.

#### geNorm analysis

**Figure 2:**
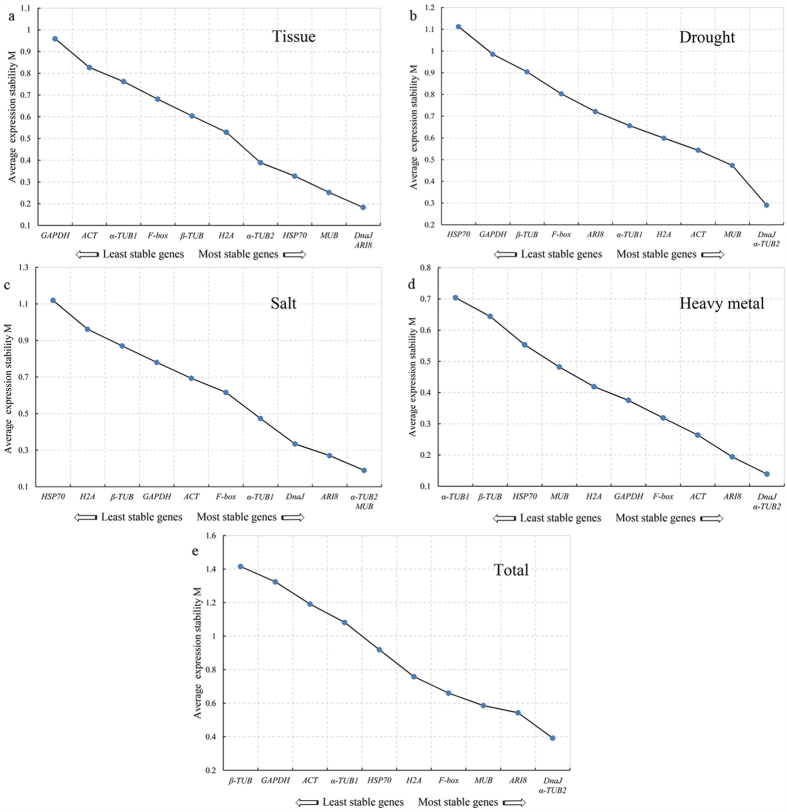
Expression stability of 11 candidate genes as calculated by geNorm. (a) different tissues, (b) drought treatments, (c) salt treatments, (d) heavy metal treatments, (e) all samples..

The stabilities of the 11 candidate reference genes of *S. matsudana* calculated using geNorm were ranked in the different tissues and abiotic stress treatments according to their M values, as shown in Fig. 2. The lowest M value indicates the most stable reference gene, and the highest M value indicates the least stable one. *DnaJ* and *ARI8* had the highest expression stabilities in the six tissues, and all the genes had M values below the threshold of 1.5 (Fig. 2a). The top two most stable genes were *DnaJ* and *α-TUB2* for drought and heavy metal stresses, and *α-TUB2* and *MUB* for salt stress (Fig. 2b,c,d). When the stabilities from all the samples were combined, *DnaJ* and *α-TUB2* were determined to be the most stable reference genes in all the samples (Fig. 2e), while *β-TUB* had the less stability.

**Figure 3:**
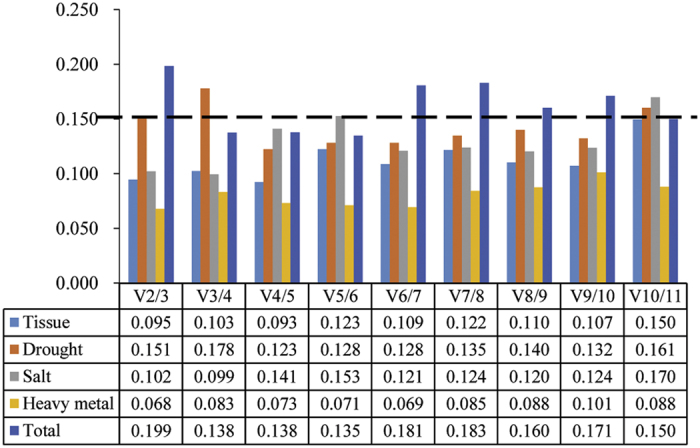
Determination of the optimal number of reference genes for normalization by pairwise variation (V) using geNorm. The average pairwise variations (Vn/Vn+1) were analyzed to measure the effect of adding reference gene on the qRT-PCR.

The pairwise variation (V_n_/V_n+1_) between two sequential normalization factors NF_n_ and NF_n+1_ was calculated by the geNorm algorithm to determine the optimal number of reference genes for accurate normalization. A cutoff value of 0.15 is the recommended threshold indicating that an additional reference gene will make no remarkable contribution to the normalization. The V_2/3_ values in the tissues, salt, and heavy metal were less than 0.15 (Fig. 3), which suggested that the top two reference genes were sufficient for accurate normalization. For the drought stress samples V_4/5_ was 0.123, indicating that the top four reference genes (*DnaJ, α-TUB2, MUB*, and *ACT*) were needed for accurate normalization. For the total samples V_3/4_ was 0.138, showing that three reference genes (*DnaJ, α-TUB2*, and *MUB*) were required.

#### NormFinder analysis

As shown in Table 2, *DnaJ* was the most stable gene (lowest stability value) in the salt and drought subsets calculated using NormFinder. For the heavy metal samples, *α-TUB2* was the most stable gene, while *ARI8* was the most stable gene in the different tissues. When all samples were taken together to determine the stability of reference genes, the three most stable genes were *α-TUB2, ARI8*, and *DnaJ*.

#### ΔCt analysis

The 11 candidate reference genes from the most to least stable expression, as calculated by the ΔCt method, are listed in Table 3. *α-TUB2* was the most stable reference gene in the drought, heavy metal, and total samples subsets. *MUB* and *ARI8* were the most stable genes for the salt subset and different tissues, respectively, and were considered the ideal reference genes.

#### BestKeeper analysis

BestKeeper determined the stabilities of the candidate reference genes based on their standard deviation (SD). Genes with SD > 1 was considered unacceptable reference genes. The genes are listed from most to least stable in Table 4. *DnaJ* was the most stable gene in the tissue and drought subsets, while *GAPDH* and *α-TUB2* were the most stable genes in the heavy metal and salt subsets.

#### RefFinder analysis

**Figure 4:**
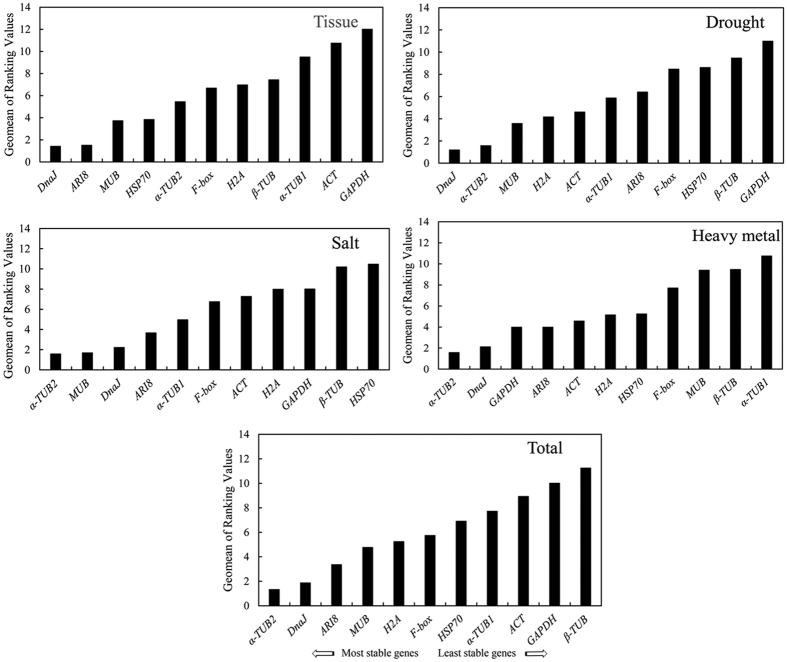
Expression stability of 11 candidate reference genes as calculated by RefFinder. A lower Geomean value indicates more stable expression.

To acquire reliable results for the expression stabilities of the 11 candidate reference genes of *S. matsudana*, the rankings of the four algorithms were integrated by RefFinder and the results are shown in Table 5. The 11 genes were ranked from the most to least stable expression by RefFinder (Fig. 4). The expression of *α-TUB2* was ranked the most stable under the salt and heavy metal stress treatments, and the expression of *DnaJ* was ranked the most stable under the drought stress treatment. Overall, the best reference gene for accurate transcript normalization in all of the samples was *α-TUB2*, which had the lowest Geomean (geometric mean) of the ranking values.

### Reference gene validation

**Figure 5:**
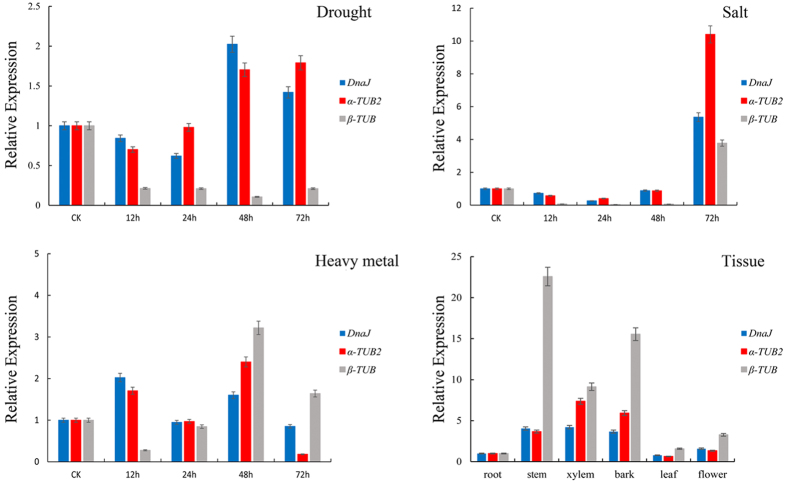
Relative quantification of *SmCAT* expression using validated reference genes .

To validate the performance of the best ranked candidate reference genes, the expression patterns of *SmCAT* (catalase) were analyzed (Fig. 5). C*AT* as abiotic stress inducible genes, are up-regulated by drought^54^, salt^55^, and Cd^56^ treatments. The *CAT* with low affinity towards H_2_O_2_ but with a high processing rate^57^, can operate through a complex networking machinery to avoid damage caused by ROS^58^. In this study, we used the most stable reference genes (*α-TUB2* and *DnaJ*) and the least stable gene (*β-TUB*) as internal controls for normalization of *SmCAT* according to the RefFinder rankings. The expression profiles of *SmCAT* were determined in different tissues and under drought, salt, and heavy metal stresses. When the stable reference genes *α-TUB2* and *DnaJ* were used for normalization, *SmCAT* exhibited similar expression trends. However, when the least stable reference gene *β-TUB* was used for normalization, the expression patterns of *SmCAT* were different from that obtained using the two stable reference genes.

## Discussion

Abiotic stress conditions including drought, salt, and heavy metals bring great losses to forestry productivity and increase the risk of environment. To guarantee sustainable forestry productivity and decrease the risk of environment, it is imperative to understand the regulation and function of the key genes under different abiotic stresses. To study gene expression variations and determine gene regulation patterns, suitable reference genes are prerequisite to accurately determine the expression levels of target genes. qRT-PCR is a reliable and accurate technique for measuring gene expression levels. Some suitable reference genes under abiotic stresses, such as *GAPDH*^59^,^60^ and *DnaJ*^10^, have been detected in plants; however, the number of reference genes evaluated is limited, especially for woody plants.

*S. matsudana* is an important afforestation and greening material in China that can adapt to harsh environments including drought, salt, and heavy metal. A good understanding of the molecular mechanisms related to abiotic stress responses in woody plants will not only help in improving forestry productivity but also help to decrease the risk of environment. A few studies have explored the ability of *S. matsudana* to withstand different abiotic stresses; however, the study of reference genes in willows has lagged behind that of other major plant species. To address this problem, we analyzed the expression of 11 candidate reference genes, five traditional reference genes (*ACT, α-TUB1, α-TUB2, β-TUB*, and *GAPDH*) and six new genes (*DnaJ, ARI8, MUB, HSP70, F-box*, and *H2A*), in various tissues, including the roots of *S. matsudana* under different abiotic stresses using qRT-PCR methods. The best and worst candidate reference genes were further verified by expression profiling of the defense response gene *SmCAT*.

We used five different statistical algorithms to determine the stabilities of candidate reference gene(s) under various abiotic stress conditions in *S. matsudana*. The results listed in Table 5 showed that, for the most parts, geNorm, NormFinder, ΔCt, and RefFinder consistently ranked the same genes as the most stable candidate reference genes. The BestKeeper algorithm is different from the other algorithms, which explains why the BestKeeper results showed the least correlation with the others^61^. Therefore, we selected the reference gene(s) determined by geNorm, NormFinder, ΔCt, and RefFinder.

*α-TUB2* and *DnaJ* were the two most stable reference genes in all the sample sets according to the four algorithms. *α-TUB2* encoding a cytoskeleton structure protein^62^ and *DnaJ* encoding a cellular chaperone have the ability to repair heat-induced protein machinery damage^63^,^64^. Our results are in agreement with several previous studies, which showed that *α-TUB2* and *DnaJ* were established as the most stable reference genes in plants under abiotic stresses; for example, in *Syntrichia caninervis* under drought, salt, and heavy metal stresses^65^, *Corchorus capsularis* under drought stress^10^, *Buchloe dactyloides* under salt stress^66^*, and Platycladus orientalis* under salt stress^67^. Normalization with multiple reference genes is an effective way to avoid erroneous data that may result from using a single reference gene^68^. In this study, two top ranked reference genes, *DnaJ* and *α-TUB2* under heavy metal stress and *α-TUB2* and *MUB* under salt stress, were appropriate for gene expression normalization, Meanwhile. Four reference genes, *DnaJ, α-TUB2, MUB*, and *ACT* under drought stress, were needed for accurate normalization. Two reference genes were found to be sufficient to analyze the expression of target genes in sorghum^62^, jute^10^, and moss^65^.

Significant differences were revealed in the expression patterns of the target gene *SmCAT* when was normalized with the two most stable genes (*α-TUB2* and *DnaJ*) compared with one of the least stable genes (*β-TUB*) (Fig. 5), The results emphasize the importance of using stable reference genes for normalization. Our findings indicated that *α-TUB2* and *DnaJ* either singly or in combination are suitable for normalization of gene expression in *S. matsudana* under different abiotic stresses. Consequently, we recommend *α-TUB2* and *DnaJ* as the most suitable reference genes for normalization of qRT-PCR expression data in *S. matsudana* under diverse abiotic stress conditions.

To the best of our knowledge, this is the first report on the identification and validation of suitable reference genes for qRT-PCR analysis in *S. matsudana* under abiotic stresses.

## Conclusion

To validate suitable reference genes for gene expression normalization in *S. matsudana* under drought, salt, and heavy metal stresses, we selected 11 candidate reference genes using four systematic statistical algorithms (geNorm, NormFinder, ΔCt, and BestKeeper). The obtained results were compared and ranked using RefFinder. Based on the gene stability analysis, we identified *α-TUB2* and *DnaJ* as the most stable reference genes for normalization of gene expression under drought, salt, and heavy metal stress conditions. Furthermore, the expression profiles of *SmCAT* validated *α-TUB2* and *DnaJ* could be used as suitable reference genes. The reference genes identified in this study will facilitate accurate and consistent expression analysis of stress tolerance genes in willows and woody plants under various abiotic stress conditions for functional genomic studies.

## Additional Information

**How to cite this article**: Zhang, Y. *et al*. Selection of suitable reference genes for quantitative real-time PCR gene expression analysis in *Salix matsudana* under different abiotic stresses. *Sci. Rep.* **7**, 40290; doi: 10.1038/srep40290 (2017).

**Publisher's note:** Springer Nature remains neutral with regard to jurisdictional claims in published maps and institutional affiliations.

## Supplementary Material

Supplementary Information

## Figures and Tables

**Table 1:** Reference genes and target genes investigated in *Salix matsudana* by qRT-PCR.

Gene	Gene description	*S. purpurea* ortholog locus	Primer sequence F/R(5′-3′)	Product size (bp)	Efficiency (%)	R^2^
*ACT*	actin	SapurV1A.0285s0180	CAGAAAGACGCCTATGTTGG	104	98.9	0.9941
			TCCATATCATCCCAGTTGCT			
*α-TUB1*	alpha-tubulin1	SapurV1A.0005s0080	GAGGATGAAGACGGTGAGGA	197	92.6	0.9995
			GAAGCAAAGGGAGACAGTCG			
*α-TUB2*	alpha-tubulin2	SapurV1A.0598s0030	ACTACGAGGAAGTCGGAGCA	205	91.0	0.9974
			CAACAAGAACGGAAGCAACA			
*DnaJ*	chaperone protein DnaJ 49	SapurV1A.0212s0110	GCACCAAATTTGAGCAGGAT	137	101.6	0.9919
			TACAAAACCCCACTGCTTCC			
*ARI8*	E3 ubiquitin-protein ligase ARI8	SapurV1A.0557s0250	GTAGACGATGCCCCAAGAAA	198	92.9	0.9997
			GGATGCCCTCAAACAAACAT			
*F-box*	F-box family protein	SapurV1A.1078s0140	CCTGCAACTGCCAGACTACA	121	97.2	0.991
			ACAAGGATTTTCCCCCAAAC			
*H2A*	histone H2A	SapurV1A.2339s0010	TTGTGCTCCTGTAACGGTGA	165	99.5	0.9979
			AACACCATTGCCCACTTCTC			
*HSP 70*	heat shock 70 kDa protein	SapurV1A.1370s0010	GTGGAGGTGATGGTGCTTCT	124	95.0	0.9940
			TGAGAGCCGTGTCAAAAATG			
*GAPDH*	glyceraldehyde-3-phosphate dehydrogenase	SapurV1A.0266s0210	CAGCTGATGAGGAATGCAAA	196	96.2	0.9931
			AGCATTGTTTGGAAGCTTGG			
*MUB*	membrane-anchored ubiquitin-fold protein	SapurV1A.2454s0040	ATTCAGTCCCAGCTGTCGTT	214	94.5	0.9919
			CGGAATTCCAGAGTGGAAAA			
*β-TUB*	tubulin beta chain	SapurV1A.1459s0040	CGAGGAAGGCGAGTATGAAG	196	94.1	0.9971
			TGAGCACACCCAGAAACAAG			
**Target gene**						
*SmCAT*	catalase	SapurV1A.0016s0660	CACCGAAGCTCAATGTTTCA	190	93.3	0.9978
			GGGCACAGAGCTTGCATTTA			

R^2^, correlation coefficient.

**Table 2:** Expression stability of candidate reference genes as calculated by Normfinder.

Rank	Tissue		Drought		Salt		Heavy metal		Total	
	Gene	Stability	Gene	Stability	Gene	Stability	Gene	Stability	Gene	Stability
1	*ARI8*	0.179	*DnaJ*	0.099	*DnaJ*	0.073	*α-TUB2*	0.234	*α-TUB2*	0.388
2	*DnaJ*	0.272	*α-TUB2*	0.145	*MUB*	0.095	*DnaJ*	0.259	*ARI8*	0.392
3	*HSP70*	0.305	*MUB*	0.278	*α-TUB2*	0.255	*ACT*	0.360	*DnaJ*	0.442
4	*MUB*	0.426	*ACT*	0.360	*α-TUB1*	0.362	*ARI8*	0.367	*MUB*	0.578
5	*α-TUB2*	0.486	*H2A*	0.525	*ARI8*	0.383	*H2A*	0.418	*H2A*	0.73
6	*H2A*	0.500	*ARI8*	0.660	*ACT*	0.777	*HSP70*	0.474	*F-box*	0.869
7	*β-TUB*	0.526	*α-TUB1*	0.771	*F-box*	0.899	*GAPDH*	0.482	*α-TUB1*	1.142
8	*α-TUB1*	0.863	*F-box*	1.015	*GAPDH*	1.018	*F-box*	0.594	*ACT*	1.279
9	*ACT*	1.037	*β-TUB*	1.369	*H2A*	1.107	*MUB*	0.669	*HSP70*	1.293
10	*F-box*	1.061	*HSP70*	1.397	*β-TUB*	1.352	*β-TUB*	0.829	*GAPDH*	1.655
11	*GAPDH*	1.514	*GAPDH*	1.487	*HSP70*	1.565	*α-TUB1*	0.861	*β-TUB*	1.755

**Table 3:** Expression stability of candidate reference genes as calculated by ∆Ct.

Rank	Tissue		Drought		Salt		Heavy metal		Total	
	Gene	Stability	Gene	Stability	Gene	Stability	Gene	Stability	Gene	Stability
1	*ARI8*	0.69	*α-TUB2*	0.95	*MUB*	0.94	*α-TUB2*	0.58	*α-TUB2*	1.18
2	*DnaJ*	0.71	*DnaJ*	0.99	*α-TUB2*	0.99	*DnaJ*	0.59	*DnaJ*	1.20
3	*HSP70*	0.77	*MUB*	0.99	*DnaJ*	0.99	*ARI8*	0.61	*ARI8*	1.21
4	*MUB*	0.78	*ACT*	1.01	*ARI8*	1.02	*ACT*	0.62	*MUB*	1.27
5	*α-TUB2*	0.82	*ARI8*	1.09	*α-TUB1*	1.08	*H2A*	0.70	*H2A*	1.37
6	*β-TUB*	0.88	*H2A*	1.10	*ACT*	1.23	*GAPDH*	0.71	*F-box*	1.41
7	*H2A*	0.89	*α-TUB1*	1.21	*F-box*	1.28	*HSP70*	0.75	*α-TUB1*	1.60
8	*α-TUB1*	1.07	*F-box*	1.25	*H2A*	1.40	*F-box*	0.75	*ACT*	1.65
9	*ACT*	1.19	*β-TUB*	1.53	*GAPDH*	1.42	*MUB*	0.84	*HSP70*	1.67
10	*F-box*	1.19	*HSP70*	1.60	*β-TUB*	1.63	*β-TUB*	0.97	*GAPDH*	1.94
11	*GAPDH*	1.62	*GAPDH*	1.61	*HSP70*	1.89	*α-TUB1*	0.99	*β-TUB*	1.98

**Table 4:** Expression stability of candidate reference genes as calculated by BestKeeper.

Rank	Tissue			Drought			Salt			Heavy metal			Total		
	Gene	SD	CV	Gene	SD	CV	Gene	SD	CV	Gene	SD	CV	Gene	SD	CV
1	*DnaJ*	0.44	2.16	*DnaJ*	0.5	2.25	*α-TUB2*	0.91	4.01	*GAPDH*	0.59	2.49	*DnaJ*	1.15	5.26
2	*F-box*	0.5	2.36	*H2A*	0.59	2.22	*DnaJ*	1.02	4.53	*HSP70*	0.67	2.77	*α-TUB2*	1.24	5.58
3	*MUB*	0.53	2.36	*α-TUB2*	0.62	2.72	*ARI8*	1.02	4.26	*DnaJ*	0.77	3.47	*HSP70*	1.26	5.46
4	*ARI8*	0.55	2.56	*α-TUB1*	0.67	3.03	*MUB*	1.05	4.25	*H2A*	0.77	2.93	*H2A*	1.31	5.1
5	*HSP70*	0.72	3.26	*HSP70*	0.78	3.35	*H2A*	1.06	4.06	*α-TUB2*	0.83	3.68	*F-box*	1.41	6.19
6	*α-TUB2*	0.73	3.51	*MUB*	0.94	3.8	*α-TUB1*	1.47	6.46	*ARI8*	0.85	3.58	*ARI8*	1.43	6.13
7	*H2A*	0.97	4.04	*ACT*	1.1	4.38	*F-box*	1.48	6.32	*β-TUB*	0.85	3.43	*MUB*	1.45	5.97
8	*β-TUB*	1.02	4.78	*ARI8*	1.2	4.96	*GAPDH*	1.62	6.5	*ACT*	0.92	3.75	*α-TUB1*	2.08	9.91
9	*α-TUB1*	1.31	7.26	*H2A*	0.59	2.22	*HSP70*	1.64	6.89	*α-TUB1*	0.97	4.44	*GAPDH*	2.31	9.87
10	*ACT*	1.51	7.34	*α-TUB2*	0.62	2.72	*ACT*	1.89	7.33	*F-box*	1.1	4.84	*ACT*	2.35	9.84
11	*GAPDH*	1.57	7.71	*α-TUB1*	0.67	3.03	*β-TUB*	2.21	8.22	*MUB*	1.22	4.83	*β-TUB*	2.62	10.54

**Table 5:** Expression stability ranking of the 11 candidate reference genes as calculated by RefFinder.

Method	1	2	3	4	5	6	7	8	9	10	11
Ranking order under different tissues (Better-Good-Average)											
geNorm	*DnaJ | ARI8*		*MUB*	*HSP70*	*α-TUB2*	*H2A*	*β-TUB*	*F-box*	*α-TUB1*	*ACT*	*GAPDH*
NormFinder	*ARI8*	*DnaJ*	*HSP70*	*MUB*	*α-TUB2*	*H2A*	*β-TUB*	*α-TUB1*	*ACT*	*F-box*	*GAPDH*
Delta CT	*ARI8*	*DnaJ*	*HSP70*	*MUB*	*α-TUB2*	*β-TUB*	*H2A*	*α-TUB1*	*ACT*	*F-box*	*GAPDH*
BestKeeper	*DnaJ*	*F-box*	*MUB*	*ARI8*	*HSP70*	*α-TUB2*	*H2A*	*β-TUB*	*α-TUB1*	*ACT*	*GAPDH*
Comprehensive ranking	***DnaJ***	***ARI8***	***MUB***	***HSP70***	***α-TUB2***	***F-box***	***H2A***	***β-TUB***	***α-TUB1***	***ACT***	***GAPDH***
Ranking order under drought stress (Better-Good-Average)											
geNorm	*DnaJ | α-TUB2*		*MUB*	*ACT*	*H2A*	*α-TUB1*	*ARI8*	*F-box*	*β-TUB*	*GAPDH*	*HSP70*
NormFinder	*DnaJ*	*α-TUB2*	*MUB*	*ACT*	*H2A*	*ARI8*	*α-TUB1*	*F-box*	*β-TUB*	*HSP70*	*GAPDH*
Delta CT	*α-TUB2*	*DnaJ*	*MUB*	*ACT*	*ARI8*	*H2A*	*α-TUB1*	*F-box*	*β-TUB*	*HSP70*	*GAPDH*
BestKeeper	*DnaJ*	*H2A*	*α-TUB2*	*α-TUB1*	*HSP70*	*MUB*	*ACT*	*ARI8*	*F-box*	*β-TUB*	*GAPDH*
Comprehensive ranking	***DnaJ***	***α-TUB2***	***MUB***	***H2A***	***ACT***	***α-TUB1***	***ARI8***	***F-box***	***HSP70***	***β-TUB***	***GAPDH***
Ranking order under salt stress (Better-Good-Average)											
geNorm	*α-TUB2 | MUB*		*ARI8*	*DnaJ*	*α-TUB1*	*F-box*	*ACT*	*GAPDH*	*β-TUB*	*H2A*	*HSP70*
NormFinder	*DnaJ*	*MUB*	*α-TUB2*	*α-TUB1*	*ARI8*	*ACT*	*F-box*	*GAPDH*	*H2A*	*β-TUB*	*HSP70*
Delta CT	*MUB*	*α-TUB2*	*DnaJ*	*ARI8*	*α-TUB1*	*ACT*	*F-box*	*GAPDH*	*H2A*	*β-TUB*	*HSP70*
BestKeeper	*α-TUB2*	*DnaJ*	*ARI8*	*MUB*	*H2A*	*α-TUB1*	*F-box*	*GAPDH*	*HSP70*	*ACT*	*β-TUB*
Comprehensive ranking	***α-TUB2***	***MUB***	***DnaJ***	***ARI8***	***α-TUB1***	***F-box***	***ACT***	***H2A***	***GAPDH***	***β-TUB***	***HSP70***
Ranking order under heavy metal stress (Better-Good-Average)											
geNorm	*DnaJ | α-TUB2*		*ARI8*	*ACT*	*F-box*	*GAPDH*	*H2A*	*MUB*	*HSP70*	*β-TUB*	*α-TUB1*
NormFinder	*α-TUB2*	*DnaJ*	*ACT*	*ARI8*	*H2A*	*HSP70*	*GAPDH*	*F-box*	*MUB*	*β-TUB*	*α-TUB1*
Delta CT	*α-TUB2*	*DnaJ*	*ARI8*	*ACT*	*H2A*	*GAPDH*	*HSP70*	*F-box*	*MUB*	*β-TUB*	*α-TUB1*
BestKeeper	*GAPDH*	*HSP70*	*H2A*	*DnaJ*	*α-TUB2*	*ARI8*	*β-TUB*	*ACT*	*α-TUB1*	*F-box*	*MUB*
Comprehensive ranking	***α-TUB2***	***DnaJ***	***GAPDH***	***ARI8***	***ACT***	***H2A***	***HSP70***	***F-box***	***MUB***	***β-TUB***	***α-TUB1***
Ranking order under total samples (Better-Good-Average)											
geNorm	*DnaJ | α-TUB2*		*ARI8*	*MUB*	*F-box*	*H2A*	*HSP70*	*α-TUB1*	*ACT*	*GAPDH*	*β-TUB*
NormFinder	*α-TUB2*	*ARI8*	*DnaJ*	*MUB*	*H2A*	*F-box*	*α-TUB1*	*ACT*	*HSP70*	*GAPDH*	*β-TUB*
Delta CT	*α-TUB2*	*DnaJ*	*ARI8*	*MUB*	*H2A*	*F-box*	*α-TUB1*	*ACT*	*HSP70*	*GAPDH*	*β-TUB*
BestKeeper	*DnaJ*	*α-TUB2*	*HSP70*	*H2A*	*F-box*	*ARI8*	*MUB*	*α-TUB1*	*GAPDH*	*ACT*	*β-TUB*
Comprehensive ranking	***α-TUB2***	***DnaJ***	***ARI8***	***MUB***	***H2A***	***F-box***	***HSP70***	***α-TUB1***	***ACT***	***GAPDH***	***β-TUB***
