# Supplementary material for: Selection of suitable reference genes for quantitative real-time PCR gene expression analysis in Salix matsudana under different abiotic stresses
Source: Sci Rep. 2017 Jan 25;7:40290. doi: 10.1038/srep40290 (PMC5264508; doi:10.1038/srep40290)
Supplement: Supplementary Information [file srep40290-s1.pdf]

Selection of suitable reference genes for quantitative real-time PCR gene expression analysis in *Salix matsudana* under different abiotic stresses

Yunxing Zhang[a,b,c,1], Xiaojiao Han[a,b,1], Shuangshuang Chen[a,b], Liu Zheng[a,b], Xuelian He[a,b], Mingying Liu[a,b], Guirong Qiao[a,b], Yang Wang[d*], Renying Zhuo[a,b*]

[a] State Key Laboratory of Tree Genetics and Breeding, Chinese Academy of Forestry, Beijing 100091, China

[b] Key Laboratory of Tree Breeding of Zhejiang Province, The Research Institute of Subtropical of Forestry, Chinese Academy of Forestry, Hangzhou, Zhejiang 311400, China

[c] School of Architectural and Artistic Design, Henan Polytechnic University, Jiaozuo, Henan 454000, China

[d] College of Plant Protection, Yunnan Agricultural University, Kunming, Yunnan 650201, China

* Corresponding authors

E-mail address: zhuory@gmail.com (R.-y. Zhuo)

1 These authors contributed equally to the work

Captions

Tables

Table 1 Reference genes and target genes investigated in *Salix matsudana* by qRT-PCR.

Table 2 Expression stability of candidate reference genes as calculated by Normfinder.

Table 3 Expression stability of candidate reference genes as calculated by delta Ct.

Table 4 Expression stability of candidate reference genes as calculated by BestKeeper.

Table 5 Expression stability ranking of the 11 candidate reference genes as calculated by RefFinder.

Figure legends

Figure 1 Expression levels of 11 candidate reference genes across all experimental samples.

Figure 2 Expression stability of 11 candidate genes as calculated by geNorm. (a) different tissues, (b) drought treatments, (c) salt treatments, (d) heavy metal treatments, (e) all samples.

Figure 3 Determination of the optimal number of reference genes for normalization by pairwise variation (V) using geNorm. The average pairwise variations (Vn/Vn+1) were analyzed to measure the effect of adding reference gene on the qRT-PCR.

Figure 4 Expression stability of 11 candidate reference genes as calculated by RefFinder. A lower Geomean value indicates more stable expression.

Figure 5 Relative quantification of *SmCAT* expression using validated reference genes.

Supplementary material

Figure S1 The melting curves, amplification plots, and sequencing peaks of 11 candidate reference genes. (a) Amplification plots of the 11candidate reference genes. (b) Melting curves of the 11candidate reference genes. (c) The sequencing peaks of 11 candidate reference genes.

Figure S2 Confirmation of primer specificity and amplicon size. (a) Specific qRT-PCR products of the expected size for each gene shown by 2% agarose gel. (b) Melting curves of the 11 candidate reference genes and the target gene *SmCAT* show single peaks.

File S1 Sequences of the qRT-PCR products for the 11 candidate reference genes and the target gene *SmCAT*

Figure S1

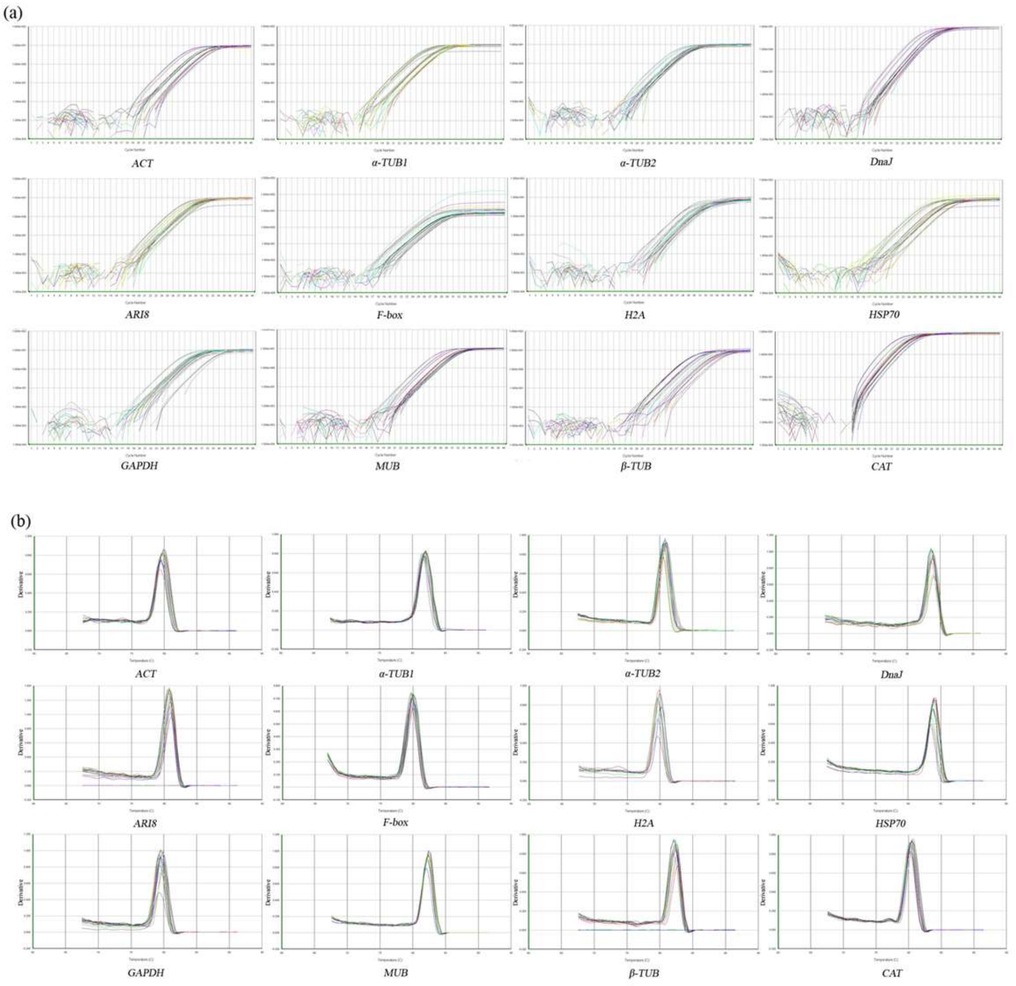

(a)
ACT
α-TUB1
α-TUB2
DnaJ
ARI8
F-box
H2A
HSP70
GAPDH
MUB
β-TUB
CAT
(b)
ACT
α-TUB1
α-TUB2
DnaJ
ARI8
F-box
H2A
HSP70
GAPDH
MUB
β-TUB
CAT

(c)

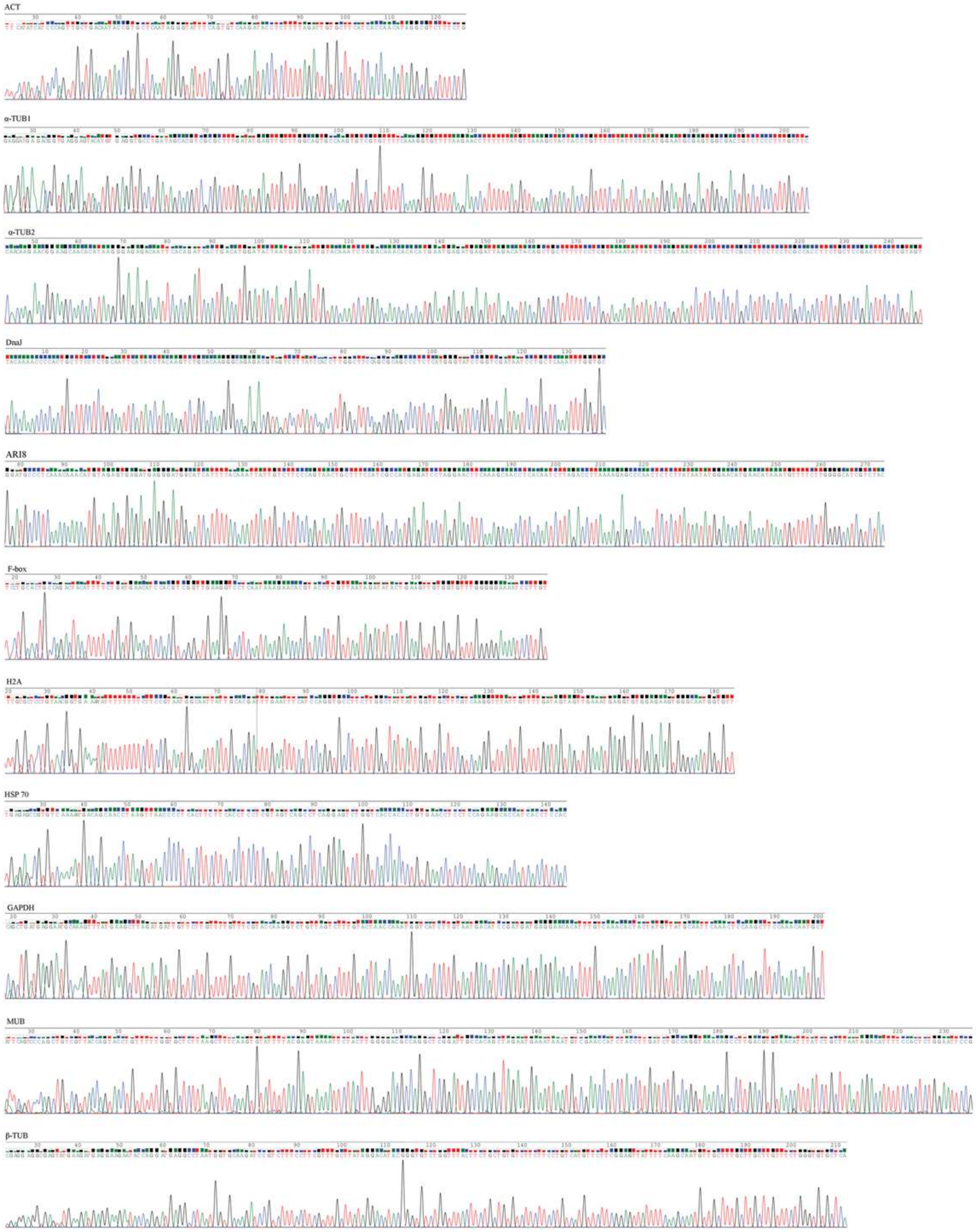

Figure S2

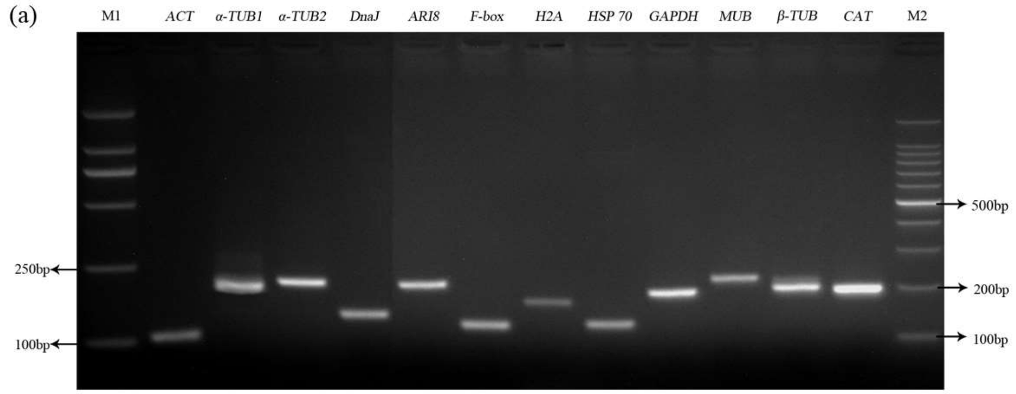

(a)
M1
ACT
α-TUB1
α-TUB2
DnaJ
ARI8
F-box
H2A
HSP 70
GAPDH
MUB
β-TUB
CAT
M2
250bp
100bp
500bp
200bp
100bp

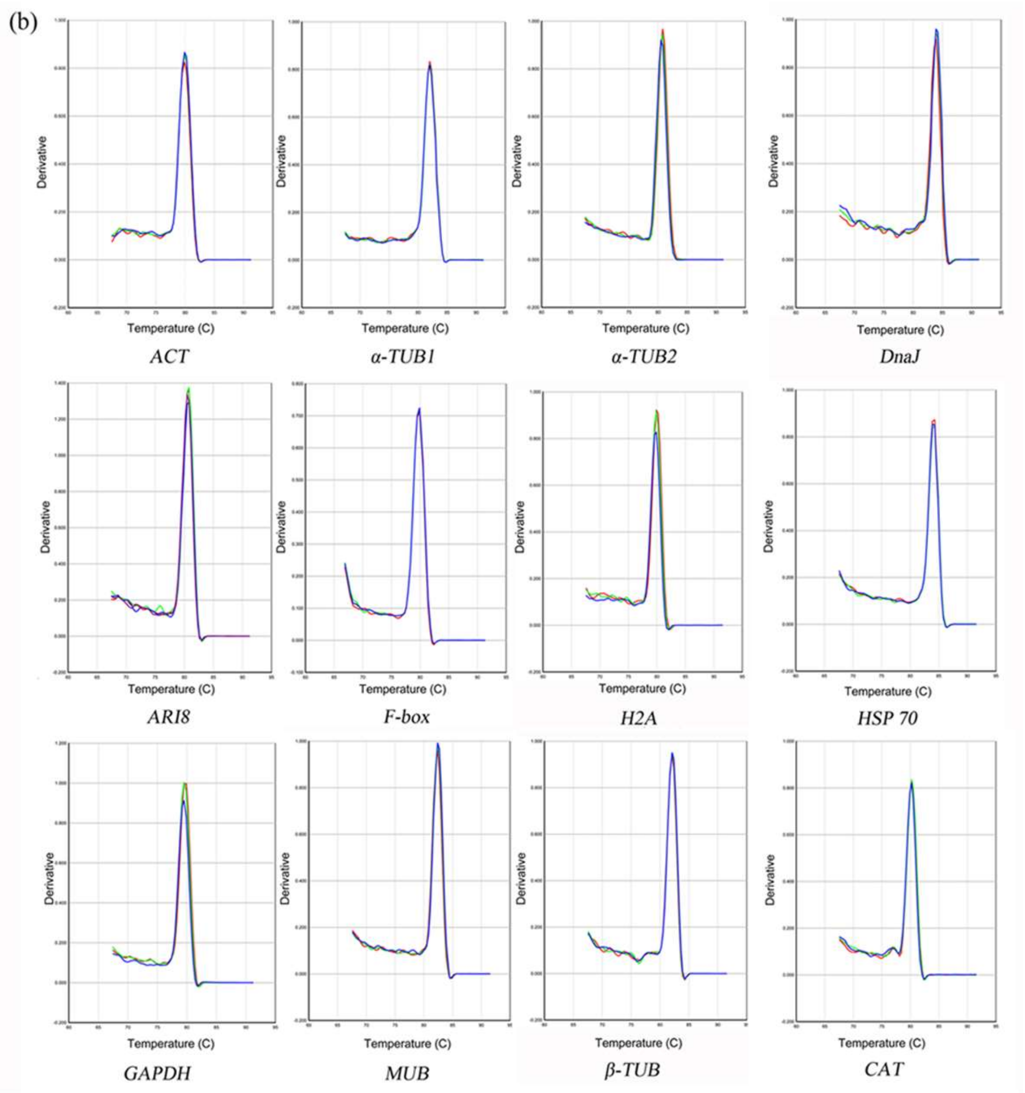

(b)
Derivative
Temperature (C)
ACT
α-TUB1
α-TUB2
DnaJ
ARI8
F-box
H2A
HSP 70
GAPDH
MUB
β-TUB
CAT

File S1

ACT

GTGTGATTTGTGCTTCATGCGAGAAAAATGAGTGATCAATGTGACTGGCATGATCTAT
CTTGTGCCCGATAAGCTGGTCGGGATTGTGTAAATCCTTAGTGTGGTGTAGGACCACT
GGCCTTTGTTTTGGTATTGGAATGAACAGAGAATCAGATTGTTGCTTTCAGTTGAAGC
ATTTTAACATTTTGTTCAAAGTTTGTTTCTTGGCGATTTGGACAAGTCTGCTTTTATTG
TTAAAGTGCAGTAATCAAGATTAATCATGGGTTTATCTCAATTAAGATTTTTTTATTAA
TGGTATAGTGCTTTATGTTCAAGACTTTGCTATGGAAATTGCACTAAAACTCTATTGG
GAAAGATCAATTTGTTGATGTGTATTTTACTGGGGTCTTCAATTTGGATTTCTTATTTT
TGCATATTGTTCTAAGACAAATCTTTACCTTGATTCTAGATGTGAGATTTTCTGAACGC
TAGTATTTGCACACATGATTTGTGTTGTGCTTGAGAAAAATGAATGACTGATATGACT
GGTGTGTTCTTTATTATGCTCTTTAAGTTGATCATGGATTTGCATTAACCTTTGGGGTG
GTGTAGGGATCATTGGCCTTTGTTTTGGCATGGCCATGAATAGAAAATTAAATCTTTT
CTATTGAAGGATGAATTTATGGTTGTTTTTTCAATATTATTGTACCTTGCTTTGATTCTT
TGCCTTGACTAGATGGCACTACTTGAATTGCAGGATCACTATAAACCATGGCTGAAG
GTGAGGAAATTCAACCTCTTGTCTGTGATAATGGCACTGGAATGGTTAAGGCAGGTTT
CGCTGGAGATGATGCTCCAAGGGCAGTCTTCCCCAGTATTGTTGGTCGGCCTAGGCAT
ACAGGTGTTATGGTTGGAATGGGGCAGAAAGACGCCTATGTTGGGGATGAGGCCCAA
TCAAAAAGAGGTATTCTCACATTAAAGTACCCAATCGAACATGGGATTGTTAGCAAC
TGGGATGATATGGA

α-TUB1

AAGTGATCTCCAGAAGGCGTCTTGATAAACGTCTTCGTCCCCGATCTCCTTCTTCAAC
AATCAGATCTATCTTCTTTTCTTTCCTTGATCAGAGAAAATGAGAGAGTGCATTTCGA
TCCACATTGGTCAAGCCGGTATTCAAGTCGGAAATGCCTGCTGGGAACTCTACTGCCT
CGAGCATGGAATCCAGCCTGATGGTCAGATGCCAAGTGACAAGACTGTTGGTGGAGG
GGATGATGCTTTTAACACCTTTTTCAGTGAAACTGGTGCCGGGAAGCACGTCCCTCGT
GCTGTCTTTGTAGATCTTGAGCCCACTGTCATCGATGAAGTCAGAACTGGAACTTACC
GCCAACTTTTCCACCCCGAACAACTTATTAGTGGCAAGGAAGATGCTGCCAACAATTT
TGCCCGTGGACACTATACCATTGGCAAGGAAATTGTTGACCTGTGCTTAGACCGTATC
AGAAAGCTTGCTGACAACTGCACTGGACTGCAAGGTTTCCTTGTATTCAATGCTGTTG
GCGGTGGCACTGGATCTGGTCTTGGATCCCTTCTCTTGGAGCGTTTGTCAGTTGACTA
TGGAAAGAAATCCAAGTTGGGTTTCACTGTCTATCCATCTCCTCAGGTCTCCACATCT
GTTGTCGAGCCCTACAATAGTGTCCTCTCAACTCACTCCCTGTTGGAACACACTGATG
TGGCCGTGCTTCTTGACAATGAAGCCATCTACGATATCTGCAAGCGCTCTCTTGACAT
TGAACGACCAACCTATACCAACCTCAATAGACTTATCTCTCAGGTCATTTCCTCCCTG
ACCGCTTCTCTGAGGTTCGATGGTGCTTTGAATGTGGATGTCACTGAATTCCAGACCA
ACTTGGTCCCCTACCCAAGAATCCACTTCATGCTTTCCTCCTATGCACCAGTGATCTCT
GCTGAGAAAGCCTACCATGAACAACTCTCTGTTGCTGAAATCACCAACAGTGCCTTCG
AACCTGCATCTATGATGGCAAAGTGTGATCCTCGCCATGGCAAGTACATGGCCTGCTG
CCTGATGTACCGTGGTGATGTTGTGCCTAAGGACGTTAATGCTGCAGTTGCCACCATA
AAGACCAAGCGTACAATTCAGTTTGTCGACTGGTGCCCCACCGGATTCAAGTGTGGT
ATCAACTACCAGCCACCCACAGTGGTTCCTGGTGGTGATCTTGCCAAGGTCCAGAGG
GCTGTGTGCATGATCTCCAACTCCACCAGTGTTGCTGAGGTGTTTTCTCGCATTGACC
ACAAATTTGACCTCATGTATTCCAAGCGCGCTTTCGTTCACTGGTATGTTGGTGAGGG
CATGGAGGAGGGTGAGTTCTCCGAGGCGCGTGAGGATCTTGCTGCACTCGAGAAGGA
TTATGAAGAGGTAGGCGCGGAATCAGCCGAGGGTGAGGATGAAGACGGTGAGGAGT
ACATGTGAGGTGCCTGATCATGTATCTATCGCACGACACTTTGATATGAGTTGTTGTT
TGGCAGTGCCAAGTGTCGTGTTTTCAAAGGTGTTTGAAGAACCTTTTTTATGTTAACG
CTGCTACATGTTTCTTATTCTATATGGAAAAGTGATGGGCGACTGTCTCCCTTTGCTTC
AGTACTTTGATCTTCATAAACATATATATTTATGTTTTTACGGTG

α-TUB2

GGCATTCATAAGAAAATAAATCTCTTCTTTTTCTCTCTTCTTTCTATCTAATTTGTATCTC
CATTGTTGCATAGAAACTCTTTCTCTGAGTTTCTTGGAGAGATAGATATATTGTTTAAGG
AGAGAAAGAGAGGAAGAAATTAATTATGAGGGAGATAATAAGCATACATATTGGACAA
GCAGGGATTCAGGTTGGGAATTCTTGCTGGGAACTTTATTGTCTTGAACATGGAATACA
GCCTGATGGAACGATGCCAAGTGACACCTCAATCGGAGCGGAACATGATTCTTTCAAT
ACCTTCTTCAGCGAAACTGGTTCAGGAAAGCATGTGCCAAGAGCTATATTTGTTGATCT
TGAACCCTCTGTCATTGATGAAGTCAGAACTGGGACTTACAGACAACTTTTTCACCCT
GAACAACTTATTTCTGGCAAGGAAGACGCTGCTAATAACTTTGCCAGGGGACATTATAC
AGTGGGAAGGGAAATTGTTGATCTATGCCTGGACCGCGTGAGGAAATTGGCAGACAAT
TGCACTGGTTTGCAAGGGTTTTTGGTATTTAATGCTGTTGGTGGTGGCACTGGTTCTGG
TTTGGGCTCCCTACTTTTGGAACGCTTGTCTGTGGATTATGGAAAGAAGTCGAAACTTG
GCTTTACCATTTATCCTTCTCCTCAGGTTTCCACGGCTGTTGTGGAACCTTACAACAGT
GTGCTATCCACCCACTCTCTCCTTGAACACACAGATGTTGCTGTTCTTTTGGACAATGA
AGCTATCTATGACATCTGCAGGAGATCTTTAGACATTGAAAGACCAACATACACCAATT
TGAACCGTTTGATTTCCCAAATTATCTCATCTTTGACAACTTCCTTAAGGTTTGATGGGG
CCATTAATGTGGACATCACGGAGTTTCAAACTAATCTCGTTCCATATCCTCGCATCCATT
TCATGCTTTCTTCTTATGCTCCAGTAATCTCAGCTGCTAAGGCTTACCATGAGCAGATTT
CAGTTCCTGAGATCACAAGTGCTGTGTTTGAGCCCTCGAGTATGATGGCTAAATGTGAT
CCAAGACATGGAAAATACATGGCTTGCTGTCTCATGTATCGTGGTGATGTTGTACCAAA
GGATGTTAATTCTTCTGTTGCCACCATTAAGACAAAGAGGACAGTTCAATTTGTAGACT
GGTGCCCAACTGGTTTCAAGTGCGGTATCAACTACCAGCCACCTACAGTGGTACCTGG
AGGTGATCTTGCTAAAGTGCAGCGTGCAGTTTGCATGATAAGCAACAACACAGCAGTA
GCTGAGGTCTTCTCCAGAATTGATCACAAGTTTGATCTCATGTATTCCAAGCGAGCATT
TGTCCACTGGTATGTGGGCGAAGGCATGGAGGAAGGTGAATTCTCAGAAGCTCGTGAA
GATCTTGCTGCTCTTGAGAAAGACTACGAGGAAGTCGGAGCAGAAGGTGGCGAGGAG
GAAGGCGAGGAGGAAGATTACTGAGATAATATTTTACGAGGAAAAAGCAGCTGTATGT
CTAATCTCATCTCATTCATGTGTGTTTGTCTAGATTTGTACAATCATCATTAGTATCCATG
TCAGTGATCTGTGAATTGTCTCTCCCTTATGTGTTGCTTCCGTTCTTGTTGAATGCTGTT
GAAGGTACGATCTTAAAAAAAAAGGGTGTCATTTTCGTTCTCTGAAATGAAACAGAGC
AGTGAAATGCAGTGTATTAGGCTATCATATCTTAGAGGCGTCAATTGTTATGGTTGCACG
TAAGAACTAAGAAATGCTATAAGAAAGTCAACATGGAGAGGTTGAGGATGATGAAAG
GAAAATTTTTCACGAGCTGGACAAAACTATGAACATCTCTTGAATCTGTATTTGTCTCT
GCTACATGTAGGAGAGCATGTTGGAGAGAT

DnaJ

GAATACTCTTATTTTGTATAGAGTTTGTTTCTCTCTCTCGACCTGTTTTTCTCTCTCACAG
AATCCCCAAACTTGAAACCCTAATTTATCGATTTCAGATCCGCTTGCAAGCTGGGTTTT
CGTCTTTTGATTTCTATTAGGTGATTTGTTATTCTTACTAAGAACCGTCAGAAAATGGAT
GGAAACAAAGACGATGCGTTGAAATGCTTGAAAATCGGCAAAGAAGCCCTCGAATCC
GGTGATCGAAGCCGTGCTTTAAAATTCTTCACCAAAGCTCGTCGCCTGGATCCTGCAAT
CGCCATCGACGATCTCTTATCTGCGGCGGAAAAGGATGAGCCTAATAAAACGGCGGCT
GAAAACATTAGTAATGGGTCCACAACAGCCACGGCCTCCAACGAATCCAAAGTTCGCC
ACAGGGGTTCATCATCATCATCATATACAGAGGAGCAAATTTCGATTGTGAGAGAAATC
AGGAAGAAGAAGAATTACTATGAGATTTTGGGATTGGAAAAGTCTTGCACTGTAGAAG
ATGTTCGAAAAGCATATCGAAAACTATCACTGAAAGTCCATCCTGATAAGAACAAGTCT
CCTGGAGCTGAGGATGCATTTAAAGCTGTTTCGAAAGCGTTTCAGTGCCTTAGCAATG
AAGAGAGCAGGAGCAAGTATGATGTTACCGGAACTGAAGAGCCTCTCTACGAGAGAC
GCACTTCCAGTCATCGTCGTCATTATAGTTATAACGATGATTTAGATCCTGACGAGATAT
TCAGGCAGTTCTTCTTTGGAGCTGGAATGAGGCCTGCTACCACCCAGTTTCGGAGCTTT
AATTTTGGAGCTGGAATGGGTGGCCCTAGAATGGATCATAATGGATCTGGCTTTAATTTC
CGTGCATTGATTCAACTGCTCCCGGTTCTTCTTATTTTCCTTTTCAACTTCCTACCGTCAT
CTGAGCCTATCTATGCCCTTTCCAGGTCCTATCCTTATGAATACAGGTTTACTACGCAGA
GAGGGGTCAATTTTTATGTGAAGAGCACCAAATTTGAGCAGGATTATCGACCGGATACC
CATGAGAGGGCTGCGCTGGAAGCCAAGGTGGATAAGGACTACGTCTCTGCCCTTGTGC
AGACTTGTAGGTATGAATTGCAGAGGAAGCAGTGGGGTTTTGTAAGGGAGACTCCTCA
TTGTGAATTGCTGCAGCAGTTTCAAGATGGGGAATTGGTGGCTTGATGGAAACTTTTAA
GGTTTAATTTCATCTACTTGTGGTTTTGGTCTTAAATACTTAGTAGTTACTCAAGGATGA
ATCATCATTGGTCTTAAATACTTAGTGGTTTTGCTTCTGACGATGTAATATTTGGTAGATT
ATGGCCTCATAAGTTCTGCTTTCACTTTCTTCTGTCACATACACATAAGCATGCAAATTC
GCAAGCAAAACTTCTTTTATATTTACTTTCGACATTTTTCCATCTTCGGGACATAGCAAT
TTATAATGGGATTCTAATCGTAATTGAATAGGCGTAGTTCCTGTATTGATGCTTGACTTCA
TGACTTAAAGCAGCCTTATAGGGCCTGGCTGAATTGACGTATTCAATTGAGGCAACTAA
CTTTTTTTACCATTACATGTGCATTTTTCCTTGTTTTCTTGAGTACAATCCGCCATATTAC
TTTAGGATTGATTTGTTGTGATTGTTCATTATCTTCTCCTCGCTGCCAATGAAGACACCC
TAAAATTCTCCACTACAAAATTCTTTGAGTTGTTGCTGCTACCTGGCTTTGCATTTGCAC
AAAAAAATAAAATAATAAGAGCAATTTTTTATTCATTCCATCTGTCCTTTTTCATAAAATA
TTGTTTCTTTTTGGCCATTTACAATTGATTAAAATGCAGTGATGTGTGGGAACTCTTGGT
TTAGAGGTTTTATTCCTTTTCTTTTGC

AR18

AATCTCTCTCTGCAGTCTCGATCGTGCTCACGAAAGAAGAAAGAAGAAATAGAAAATT
AAAAAGAAGAAGAGAGAGCAGCTACTCCTTAATTAATTAATTTTGAGCACTCAGATTT
GTGGCCTAATAATCTCTATCTATCAATCGATTTCTCCCGCAGCTTGCGCTCATAAACCCT
AGTAAGAATTTGCTTGTTTGTCCTGCTCCAGTGTAGGAGGGCAGGGAGAGACAGGAG
AAGGAGAGGATTGGTGGTTGAAAATTGGGAAGGGTGAGTTTTGGTAAGCGGTGGAAA
TGCCAGAGGAGGAGGAGTTAGTTGAGCTCAAGTTTAGATTGTATGATGGATCGGATATC
GGTCCTTTTAGGTATTCACCTGCATCCACTGTTGCTATGCTCAAAGAAAGAATCGTCGC
CGACTGGCCTAAAGATAAAAAAATTGCACCCAAGGCAGCAAATGATGTCAAACTGATA
AATGCTGGGAAAATCTTGGAAAACAACAAGACCGTTGGCCAGTGTAGGGCTCCTTTTG
GGGACCTCCCGAAGGGGGTCATCACCATGCATGTTGTTGTCCAGCCATCTTTAGCAAA
AGCAAAAGCAGAGAAGAAGGTAGACGATGCCCCAAGAAAACATTTCTGTTCATGTTC
CATATTATAAGAGAGTGGGGCTCCTTTAAGGTCTAAGATTGTGAGGTTGCTTTGAAGTT
CCTAGGATCTCATGGCCATGGAAAGCAGTTACTGGGAAGACAATAATTTGTAAAATGAT
GCCATCCCTCATCTCACTCTACATGTTTGTTTGAGGGCATCCCTGTGTAACAGTGTGCT
GGTGATGTATTTACACTATAAATGGGTGTTTATGTATTTTTTATAGCTGCATGTCTGCACT
CTCATTTTATATTAATTTTATACAAGGCTTACGGGTTTGATCTTCTCTGAAATCCTATAATT
ATGTACCCACTGGTTGAGTACCGATGTGCTGTTATTTGTGAGACAATTCGATAATTTTGC
ACTTGCAGCTTGTGAACACAAACAAGGAATTGGATGCACATTTTCTGTATTCC

F-box

CTAAGCACACACTCTTGTTACTATCTCCAGGCAGCAAACAAGCAAAACCAAGAAACA
ATAACTAAACCACATGATAATCAGAATTTAAAGAGAAACAGACATGGGGCTGGAATCA
GTGGGAGATCTAGCTCTCAACACAATCTTAACGAAACTAGGTCCAGAAGAGACAGTA
AAAGTATCATGTGTCAGCAAAAAGTTCAAGGATTTAGCCGCAGAGGAATCTCTCTGGT
CATTATTTTGCCGTCAAGATCTTGATCTTTCTGCTCCTCTTGATCATCATGGCAATCCTC
TACCTTCATTTAAGGCAACTTATAAGTTATGGAGAGAAGCCTTTCATATGTATCCTTGG
CCCCTTGTAAAGCGAGTTAAAAGTTGTTGGGACAGACTCACAATCTGGTTGACCACA
AACTTTCCTGAAGCTAAGGCTACCCTAGGGAAGGGTGCATCAGAAGGTAAGATTGAA
GAGCTGGAAAAAAAATTGAAAGTTAAGTTGCCTCTTCCCACAAGACTCCTTTACCGC
TTTCATGATGGTCAAAATTTACCAAGCAAAAACCAGTCAAGTGGTATGGCTGGTTGTC
CATTGGGACTGATAGGTGGCTACTGTTTTTATAATCACTTGGTTAATGTCTACTTATTAC
CACTAGATGAGGTAATCTTTAAATCACAGAAACTAGTGCGGCACTTGAACTTACCCAA
TACATCCAAGTATATTGTTGTGGCTGCTTCATCCTCACGCATTGGAAAGTTTTTCTTCCT
TAACTGTTCTGATGGCCAACTCTATGTTGGGACCCATAATCTACCAACAGATGCAGAA
ATGATGCCATGTGTACCTCAGGCATTGATTAGTCCAGTCCATGATTTCAACAATGACCA
ACAACAGGATGCTATGTTGTTGTGGTTAGAAGAACATGGTCGTCGCCTGCACAATGGC
ACTATCAAAGTTCTGGGTGAAGGAAATATGAAAAGCATCTCTCAGTTTCCAGAAGAAT
CTCCTCTTTGTTCAACTGCTGTAACCAGTGGTGTAAAGGTTCGTGCTTCTGCTGTTTTT
GTGCCAGAGGCTGCTGATTTGCAAGATAATTCTAGAGAATATGTGTTTGCTTATTCAAT
CCGCATGTCCCTTCAACCAGAAGGATGCATCATCAATGGAATGCACTTCGGCTCTTGC
CAACTGCACATGAGGCACTGGGTTATCAGTGCTAATGATACCGTTGTATCTAATGTCAA
TGCAGAGGCTGTGATAGGCAAGTTCCCACTCTTGTGTCCAGGCGAGAAAGAATTTGT
TTATGAGAGTTGTACACCTCTGCCAACTTCTACTGGCTCTGTTGAAGGTTCTTTCACAT
TTGTCCCTGGCAGATTGACAGATCCAAAAGGAAGTCCATTTGAAGTTGAAGTCCGTC
GGTTTCCCCTGCAACTGCCAGACTACATTTTCTGATGAACATCCACGTCGGTTGAAGG
TCCTCAATAAAGAACACGTACCTTGTTAATAGATATACTGAAGTTGTGGTGTTTGGGG
AAAATCCTTGTTACATTTCATGGATTGCCTGTTGTGTGTGAGCCACTGATTTCTCAACT
ATGAGTGCCTCTGGCTTTGAAAGAAAACAGTCCATTTCTACCAAAGCCAGAGGCACT
CATAGTT

H2A

CCTTAAAGTAGGACCAAGCTAGGCCTGAAGGCCATATTTTCTAAGGCCTAGCCCATAAT
AATCCAAACTGCCAGTTTTCTTTCTCCTATTGGGCCCATATTCCCGAAACCCTTCAAAAT
TCGAAATCCGATTGTCAATTCACCAATCCCAACAAAAATTTCCCAAAAACAAAACCAA
GCGGTTAGCAGAATCTCCACTGTCCTTTCATTTCCAATCCAACGGCCAGAGATATCGAT
CCGCGTGCTCCACTATCATTGGCCCACCCCATTTTCCCTTCTCCTATATAAACCCCAAAA
AACAGATAATTATTTCTCAATTATTCTATTGCCCTCGCGTTTTTCCTCTCCAAAAAAGAA
AGGGCGCTTCACTTGATACGGTTTTCGAAATTTTTGTTTGACAAGAATTAATCAATGGC
TGGTAGAGGCAAAACCCTAGGATCTGGAGCCCAAAAGAAGGCTACTTCAAGGAGTAG
CAAGGCTGGTTTGCAATTTCCTGTGGGTCGTATCGCTAGGTTCCTGAAGGCCGGCAAGT
ATGCTGAGCGTGTAGGCGCCGGCGCTCCTGTTTACCTTGCTGCTGTTCTTGAATATCTT
GCTGCCGAGGTTCTAGAATTGGCTGGAAATGCAGCAAGAGACAACAAGAAGACCCGT
ATTGTGCCACGCCACATCCAGCTAGCAGTTAGGAATGATGAGGAGCTTAGCAAGCTTC
TTGGTGATGTTACAATTGCTAATGGAGGTGTCATGCCCAACATTCACAACCTTCTCCTT
CCAAAGAAGGCTGGCTCCTCTAAGGCCCCTGCTGATGATGACAGTTAAATTTGAAGCA
ATATATTTCCGTTCCTGTGATTCACTACGATATCAAGTCTTTTTAGTTTAATTTTTTCTTTC
AGTTATTAGGATTTAGAGTTCTGTGTTCATGTAAGGGGGGTTGTTTTAGTAGTTAGTGAT
GTGAATATAAATTGATCTCTTTGTGCTCCTGTAACGGTGAAAATATTTTTTTTCTTCCGTA
ATGGCAATTATTGCACGATTTGAATTTCATCCAGGTGCCTTCTTGGCTATTATTGGTTGCT
TCGTCAAGGTTTATTGTTTTGATAGTAGTTGAAATGAGGTGTGGAGAAGTGGGCAATGG
TGTTTTGGTTGATATTTGGTAGGCAGGAATACAAGCAGATCTCCTTAATTATCTTGCACG
AGCTTGGGCAGACGATATACTTTCTGCATGGATGAAATGATTTCTGATATCATTCAAGGT
TTAAACCTGTACAACCTGTTGCCAGATGATCTCTACGTTTCTGTTTCAAGAAATAGTTTT
GCAGTGATTGTAGTGAATTGCGGTAAACTGAGGAATACAGTAGCGCGCAGTCGTAAGC
TTTTTCGCTTGTTGAATTAAATTATCTGTAAAAGTCGATTGTCTCTTAGGAAGAAAGGG
TGTATTTCTGTATCTAAGGCTTGATTCTATCTGATATATTTCTGTATCGACGGCGATGGCA
GTTTTGCTAGCAATTTTTCCTGTTTAGAGCAGTTCATTTCTTGAATGATTCAAGCAAAGC
TTATGAGAATGCTTCTTCACCAGGGAGTTTGACTCTTACTGGTTCACGGGAGATTTTAC
GTGTCCCATGTTACTCGAGTCAAACATGCGGATCTCCCCAGCTT

HSP 70

GAACAATCGTTTGCGAGTTGCGCCACATCAAAGGCCTTTGCTCCACAAAACCCTAAAC
CCTTCTTTATAAACCCTCAAAACACTGTCCTCGTGTCGTTCTACGCTTCCACTCCAATTT
AACTCGTAGATCTCTTCACTTATGGCCACAGCTGCTCTCCTCCGCTCTCTACGACGCCG
TGACGTCGCCTCCGCTCCTCTTTCAGCCTACAGATGCTTGACCAATAATGTGAAGCCAT
CGTGGGCTCCTTCTAATTTCAGCCAAAACTGGGCTGGTTTGTCCAGAGCTTTCAGTGC
AAAACCTGCATGTGGAGATGTTATTGGTATTGATCTGGGTACAGCAAATCCATGTGCTG
CTGTTATGGAAGGGAAGAATCCCAAAGTTTTTGAGAATGCTGAAGGATGTAGGATAAC
CCCTTCAGCTGTTACCTTCACCCCAAAGATAGTAGCCGGGATCTTTGGAAATAGCCTGA
GCAAGGGAGTAAACCCAGTTGAGGTGGTGGCTATGGGAGCTGCAATTCAGGGTGGAA
TTTTACGTAGAGATGTCAAAAAATTGTGTCCCCTTGATGTCGCTCCCTTGTCACTTGGTA
TCAAGACACGGGTTTTCTCCACAGGGACTGACAACAAGACCCAGGTTGCGGGTATTCC
ACCAACTCCCAGGGGCATGCCTCAGATTGAGGTGACCTTTGATATTGATGCCAATGGTA
TTGTTACTGTTTCTGCCAAGGACAAAGCCACTGCCAAAGAACAGCAGATCACCATACG
TTCATCTGGAGGTCTTTCAGAAGATGAGATTGAGAAGATGGTCAGGGAGGCCGAACA
GTTTGCTCAGAAAGATCAAGAGAGAAAGGCCTTGATTGATATCAAAAATAGTGCGGAC
ACCACTATCTACAGCGTGGAGAAAAGCCTGAATGAGTATAGGGAGAAGATTCCCTCTG
AGATCGCAAAGGAGATTGAAGATGCTGTTGCAGATTTGAGGAAGGCAATGGGTGGAG
ACAACGTCGATGATATCAAATCTAAACTAGATGCCGCAAATAAAGCGGTTTCAAAGATT
GGAGAGCACATGTCCAAGGGCAGCAGTGGTGGAGGTGATGGTGCTTCTGGAGGAGGT
TCACAGGGTGGTGACCAGACTCCTGAGGCTGACTACGAGGAGGTGAAGAAGTGAGGG
GTTAACTTAGGTTGCTGTCATTTTTGACACGGCTCTCAATCATATTTGAAACATCTTTTT
TAACTATATTTGCACTTTAGGATATACGTTTCCTAGCGGTTTTGTATCTAAATGGTGAGAT
TAATTTGAATGTATTACTTCTGTTCATCCGCCAATGGATGCCGAGGACCTTGCAATCATA
ATATCAATAAACCATTTTTTTCTGGGAGGAGATTGTTTGTTCATCTGACACTGCCTATCA
ATGACGTGGCGGCGGAGG

GAPDH

AAAATACTAAGGAATTGTAAAATGAGAATAACTGAAAAAACGAGTTGAACTATATAT
ATAGCCAAGAAATTGCTCTCTTTAGCTTTAGTTGCAGCCATGGCTACCCACGCAGCTC
TTGCCTCTTCAAGAATCCCTGCCAATACAAGACTTCCCTCAAAGATCAACCACTCTTT
CCCCACTCAATGCTCCTTAAAGAGGCTAGAAGTAGCCGAGTTTTCTGGGCTTCGAGCC
AGTTCATGTGTAACCTATGCCAAGAGCGCTGGTGAGGGATCCTTCTTTGATGTGGTGG
CTTCCCAACTTGCTCCAAAGGTTGCAGTTTCAACTCCTGTCAGGGCAGAAACTGTGGC
CAAATTAAAGGTTGCTATCAACGGATTTGGACGCATTGGCAGGAACTTCCTGCGATG
CTGGCATGGTCGCAAAGACTCTCCCCTTGATGTAATTGTTGTCAATGACAGTGGTGGT
GTCAAGAACGCTTCCCACTTGTTGAAATACGATTCAATGCTTGGAACTTTCAAAGCAG
AGGTGAAAATTGTGGACAACGAGACCATCAGTGTTGATGGCAAGCTCATTAAGGTTG
TTTCCAGCAGAGACCCTCTTAAGCTTCCTTGGGCTGAGCTAGGAATAGACATTGTTAT
TGAGGGAACCGGAGTTTTTGTGGATGGTCCTGGTGCTGGGAAACATATTCAAGCTGGT
GCCAAGAAAGTTATAATCACTGCTCCAGCCAAAGGTGCCGATATTCCAACCTATGTTG
TTGGTGTTAACGAAAAGGACTACGGCCATGAGGTTGCCGACATTATAAGTAATGCTT
CTTGCACCACAAATTGTCTGGCTCCCTTTGTGAAAATCCTGGATGAAGAATTCGGCAT
TGTCAAGGGAACAATGACAACAACTCACTCCTACACTGGAGATCAGAGGCTCTTGGA
TGCTTCACACCGAGACTTGAGGAGAGCCAGGGCTGCAGCATTGAACATAGTCCCAAC
AAGCACTGGTGCAGCCAAGGCTGTATCTCTTGTGCTGCCCCAGCTCAAGGGCAAGCT
CAATGGCATCGCACTCCGTGTCCCGACACCCAATGTTTCAGTTGTTGACCTTGTTGTG
AATGTTGAGAAGAAGGGCATTACAGCAGAAGATGTCAATGGAGCCTTCAGAAAGGC
GGCTGGGGGCCCATTGAAGGGTGTATTGGATGTGTGTGATGTTCCTCTTGTGTCTGTT
GACTTCCGATGCTCTGATGTTTCCTCAACCATTGACTCTTCATTGACCATGGTCATGGG
AGATGATATGATCAAGGTTGTCGCCTGGTATGACAATGAATGGGGATACAGCCAAAG
GGTCGTCGATTTAGCACATCTTGTAGCCGGAAAGTGGCCAGGAGTGGCTGCAGCAGG
AAGTGGAGACCCATTGGAGGATTTCTGCAAGACAAACCCAGCTGATGAGGAATGCAA
AGTTTATGAAGCTTAGAAGATTGTTCTTGTTTCTTTTTTTGTTTCCTTTCGTACCAAGGT
CTGTTAGTCTTTGTACTAACCAAATGGTCATCTTGTAATGACATCCGATGATGAGGAA
CACATTTGTCAAACACTACTATGTTATGCAATTCAAACTCCAAGCTTCCAAACAATGC
TTGTTATGATTTATGACGATCTCTGTTAAAGAATAACAAATCA

MUB

CAGAATCATAAAGCATTTCTTCCAGTGCGTCCCTAACTTTCCTCTCTTCTCTCCTT
TCGCCAAATCTCCATTTCATCACTCCGTCTTCCACTAAATTACATTCTCTACCCTC
CAACCTCTCCTTCTCGCTCTCTGATCATCTATTTCCAATTACATATAGATTGTATCA
ATTTGCCAGGGTTCAGAATCAAACAAAACTCAAAATCAATGGAGTCGGAGGATG
AGTTCGACATGCATGACGCGGCGGCGGAATCTGCGGAGGATGATTTCTACAGCG
GCGGAGAAGAAGATGGATTCGACAGCGACGATGCTGACGTCGCCGATTACGAGT
TCATCGACAACGACTCAGATGATTCCGATGACCTCATCTCTCATCGCCACCAGCA
AAACTATACAATTTTGAGCGAAGGAGATATACGACAGCGACAAGATGATGATATT
ATGAGGATAGCTACCGTGCTTTCCATTTCAAAGGTCGCCGCCAGTATACTTCTCC
GGTACTATAACTGGAGTGTGAGTAAAGTGCATGATGAATGGTTTGCTGACGAGG
AAAAAGTTCGAAGGGCTGTTGGTTTATTGGAGGAACCGGCTGTTCCATTTCCGG
ATGGTAGAGAAATGACTTGCGGTATTTGTTTTGAAACTTATCCTTCTGATAGATTA
CGCGCTGCTGTTTGTGGTCATCCATTTTGCAATTCATGCTGGGCAGGTTACATCA
GCACAGCCATTAATGATGGTCCTGGATGTTTGATGTTGCGATGTCCTGATCCATCT
TGTAATGCTGCTGTTGGTCAAGATATGATTAATTTACTGACTTCTGATGAAGACAG
TAAGAAACATTCTCGTTATTTCATAAGATCTTATATTGAGGACAATAGAAAGACCA
AATGGTGCCCTGCTCCTGGCTGTGATTATGCTGTGGATTTTATTGTTGGTAGTGGG
AGCTATGATGTTATCTGCCGCTGCGCATACTGCTTTTGCTGGAATTGTACCGAGG
AAGCTCACCGCCCTGTTGATTGTAGTACTGTGGCCAAGTGGATACTGAAGAACA
GTGCGGAGTCTGAAAATATGAACTGGATATTGGCTAATTCTAAGCCTTGCCCAAA
GTGCAAGCGGCCGATTGAGAAAAACCAAGGCTGTATGCATATTACATGCACCCC
ACCTTGCAAATTTGAGTTCTGCTGGCTTTGCCTTGGTGCATGGTCAGATCATGGA
GAGAGGACTGGTGGTTTTTATGCATGTAACCGCTATGAGACTGCAAAGCAAGAG
GGAGTGTATGACGAGGCTGAGAAGCGAAGAGAAATGGCGAAAAACTCTCTAGA
GAGATACACTCATTATTATGAAAGATGGGCAACCAACCAAGCATCTCGGCAGAA
GGCACTGGCAGACCTACAGCAAATGCAAAATGTGCATCTTGAGAAGCTGAGTGA
CACACAATGCCAACCTGAGTCACAGCTGAAGTTCATATTAGAGGCCTGGCTACA
GATAGTTGAATGTAGGCGAGTTCTAAAATGGACCTATTCCTATGGATACTATTTGC
CTGAGTATGAACATGCCAAGAGACTGTTCTTTGAGTATTTGCAAGGTGAGGCCG
AGTCTGGATTGGAGCGACTGCATCAATGTGCAGAGAAGGAGCTTCAAATTTACC
TAAACGCAGAGGGCCCATCAAAAGATTTAATGAGTTCCGCACTAAACTTGCTG
GATTGACCAGTGTAACTAGGAACTACTTTGAGAATTTAGTCCGGGCTTTAGAGAA
TGGTCTATCAGATGTGGATTCCCATGGTGCATGCAGCAGGATGGCAAGCTCGAA
GAGCTTGGGAGGTGGTAGCAGTAGGGCAAGAGCTGGTAGAGGCAAGGGCTCAA
CATCTAGGTCAAGTGGACCTGGTAGAAATATTGATGATCCCGGGCACTGGTCCTG
TGAACATTGCACCTTTGCAAATATAAAGCCGGCCACCATTTGTGCGATGTGCCAG
CAACGACGGTAAATCTCATTCCCTACATTGATTGGCCACCTGGGCGAGTCACGGG
TTTGGCCTCTATGGCTGTCCAGTTGTAACAAGGAAGGAAGGCCTTTAAGAACAT
TGCCGGGGAGAGTGCAAAAAAGGGCTATTGTAATCTCCTGGTCTGCAAAAAACC
TGAGCTGCAACAACAAAGGAGAACAGGAAAGGGTGGGAAAGGGAAAGGAAAC
TCTTCTCGTTGTTTGTTGTCTTGAATCAGCTGTATATAGTTCTCGTCATCACTTCCA
TGCTTACTTATAAGGGTCCATGAAAGTGCATTCAGTCCCAGCTGTCGTTACCAGT
ACCTGTTTTTGGTGCTCTTAAGCTTTCAAGTGTATTTTACGAGTAAAATTCTACTT

GGGGACGCCAGGCTCGGATTGCCACAGCTAGAATGAAATAAATGTCGAACCATC
TACCTTGATCTGCCAGGTAAACAGCCTTGACGTGTAACATTTATCTGCTTAATAGA
CATTTTCCACTCTGGAATTCCGTCCATGCCTCTTCCCTAACCTTTTCGATTACTTG
ATGCCCTGTTTTTTTTTGTTTTTCCAATTCAACATGTTTGTTGTGAAAAAATAAAC
TGCTTCTGCTAATGTAATGCTGTTGCAGAAAGTAAATTAAGTTGGACTTGAGAGT
CGTGGAAGGAAAACCCGGGCTGATGGGTATACTGTATTCCAATCGTACCACGTTT
ATCATCAATATTTATTTAAAACAATACGCTTGCTTTG

β-TUB

GGATCCCCAACAATGTCAAGTCTACTGTCTGTGACATTCCTCCTACAGGCTTGACAATG
GCTTCCACTTTCATTGGCAACTCCACATCAATCCAAGAGATGTTCCGAAGAGTTAGCGA
GCAGTTCACTGCCATGTTCCGCAGGAAGGCCTTCTTACATTGGTACACTGGAGAGGGA
ATGGATGAGATGGAATTTACAGAGGCTGAGAGCAACATGAACGATTTGGTCTCAGAGT
ACCAGCAATACCAGGACGCAACTGCTGACGAGGAAGGCGAGTATGAAGATGAGGAAG
AATACCAGGATGAGGCCTAATGATGCGAGATATATGTCTTTCCTTCGTTTGCTTATAGGA
TATACGGGTGTCTGGCATACTTCTGCTGCTGTGTCTTCTTCCTGTCATGTTCTTCGGAGT
TATTTTCAAGCAATGTTGCTTTGCTTGCTTGTTTCTGGGTGTGCTCATCTTTTAAATTGG
TTTTGTTTTCTCCGCAGCTCACAACGCATCGGGCTTGAATGCTGTTGTCTCCAAAACTA
GTAGTTAAACTTTAAATTGCACCAGCTCGGTGAAAATTGTCTTTCTCTTTCATATCCTTG
CACTAGTCCATGTTTTAGGGGAAGCTCAATATTGGAAAAACACTCCAATTTTTCAGAAA
AACAGAAAACAGAGTTTCTTGTCCATGTTCATAATTCTATTTTGAAATGGGCTGTAGAA
TAATTATGGTTACAAGAGAGCTCAACTATCCTTGTGTTTGAAAGACTAGTGCATGG

CAT

CCGCTTTGATTGACCTGTGATTACCACACTTCTCTCCCAGTCTCTCTCTCCTCAACTAC
CACCTGAGTTCTTGTTTTCCCTCTTTCCTCCTCCAAATCCTCTCCTCTCTCCTCCATGGA
TCCCTGCAAGCACCGTCCATCAAGCGCTTTCAACACTCCATACTGGACTACAAATTCT
GGAGCTCCGGTTTGGAACAACAACTCGTCTTTGACCGTTGGATCCAGAGGTCCAATCC
TCCTCGAGGATTACCATCTGGTGGAGAAGATTGCCAATTTTGACAGGGAGAGGATTC
CAGAGCGTGTCGTCCATGCTAGGGGAGCCAGTGCAAAGGGTTTCTTTGAGGTTACCC
ATGATATCTCTGGTCTCACATGTGCTGATTTTCTCCGGGCCCCTGGAGTGCAGACACC
TGTCATTGTCCGCTTCTCCACAGTTATCCATGAGCGTGGCAGCCCTGAAACCCTGAGG
GATCCACGTGGATTTGCAGTGAAGTTTTACACCAGAGAGGGTAACTTTGATCTTGTGG
GAAACAATTTCCCTGTCTTCTTCATCCGTGATGGGATGAAATTCCCAGACATGGTGCA
TGCCCTTAAGCCCAACCCCAAGTCTCATATTCAGGAGAACTGGAGGATTCTTGACTTC
TTCTCCCACCATCCTGAAAGTTTGCACATGTTCTCCTTCCTATTTGATGATGTGGGTGT
GCCACAAGATTATAGACATATGGAAGGCTCTGGTGTTAACACCTACACATTGATCAA
CAAGGCTGGAAAAGCCCATTATGTGAAATTTCATTGGAAACCTACTTGTGGTGTGAA
ATGTTTGTTGGAGGACGAGGCAATTAAAGTAGGAGGCACAAATCACAGTCATGCTAC
TCAGGATCTATATGACTCCATTGCAGCTGGCAACTATCCTGAGTGGAAACTTTTCATT
CAGACAATTGATCCTGACCATGAAGCCAGGTTTGATTTTGACCCACTTGATGTAACAA
AGACCTGGCCTGAGGATATCTTGCCCCTGCAGCCAGTTGGTCGCTTGGTCTTGAATAA
GAACATCGACAACTTCTTTGCTGAAAATGAGCAGCTTGCTTTCTGCCCTGCTATTGTG
GTTCCTGGTATTTACTATTCAGATGACAAGCTACTCCAGACTCGAATCTTCTCCTATTC
TGATACCCAGAGGCACCGTCTTGGACCAAACTATCTGCAGCTTCCTGCTAATGCTCCC
AAGTGTGCTCATCATAACAATCACCATGAAGGTTTCATGAATTTCATGCATAGGGATG
AGGAGGTACTCTCTCCACAGACTACTTTAATCAGAAGCTCAGCTACTTCACCTTCTGT
TTAAATTAATCATTTCGCTCTTCTTCATCTATGTTGGGGTTATTTCTGTGTCCTCTTGCC
TTTGCTTGTAATTTTTGTATTCTTCAGGTCAACTATTTCCCATCAAGGTATGATCCAGT
TCGCCATGCTGAGAGTTTCCCCATTCCTCCTGCTGTCTGCAGTGGAAAGCGTGAGAAG
TGCATCATTGAGAAGGAGAACAACTTCAAGCAACCTGGAGAGAGATACCGATCCTGG
GCACCAGACAGGCAAGAACGATTTATTTGCCGATGGGTTGATGCCTTATCTGACCCAC
GCGCCACACATGAGATCCGCAGCATCTGGATCTCATACTGGTCTCAGGCTGATAAATC
TCTGGGTCAGAAGCTAGCATCTCGTCTCAACGTGAGACCAAGCATATGAAGATGACA
CCGAAGCTCAATGTTTCAAATTGCAGAAACTGAGTAAACGAAAGGGGATTGCCGGAT
TGCAAGCAGAATATCAAATGCTAATATTTACTAGCTTTGCTGTGCCATAATAGTGAAT
CAAACTCGCATGTTTATGTGACTTCTACTCTTTGAACAGACATTCTTCTCTAATAAATG
CAAGCTCTGTGCCCCCAGGCTTAATGTAAAATTCTAATCTGCTGCGTGG
